# Antigen rapid diagnostic test monitoring for SARS‐CoV‐2 in asymptomatic and fully vaccinated cancer patients: Is it cost‐effective?

**DOI:** 10.1002/cam4.5537

**Published:** 2022-12-30

**Authors:** Mario Caccese, Anna Maria Saieva, Valentina Guarneri, Sara Lonardi, Massimo Cacco, Vanna Chiarion Sileni, Michele Gottardi, Eleonora Mioranza, Francesca Bergamo, Antonella Brunello, Vittorina Zagonel, Patrizia Benini

**Affiliations:** ^1^ Department of Oncology, Oncology 1 Veneto Institute of Oncology IOV‐IRCCS Padua Italy; ^2^ Medical Direction Unit Veneto Institute of Oncology IOV‐IRCCS Padua Italy; ^3^ Department of Oncology, Oncology 2 Veneto Institute of Oncology IOV‐IRCCS Padua Italy; ^4^ Department of Surgery, Oncology and Gastroenterology University of Padua Padua Italy; ^5^ Department of Oncology, Oncology 3 Veneto Institute of Oncology IOV‐IRCCS Padua Italy; ^6^ Hospital Health Professions Unit Veneto Institute of Oncology IOV‐IRCCS Padua Italy; ^7^ Melanoma Oncology Unit Veneto Institute of Oncology IOV‐IRCCS Padua Italy; ^8^ Onco‐Hematology Unit Veneto Institute of Oncology IOV‐IRCCS Padua Italy; ^9^ General Directorate Veneto Institute of Oncology IOV‐IRCCS Padua Italy

**Keywords:** antigen‐rapid‐diagnostic test, cancer patients, COVID‐19, SARS‐CoV‐2

## Abstract

**Background:**

Routine testing for cancer patients not presenting COVID‐19‐related symptoms and fully vaccinated for SARS‐CoV‐2 prior to cancer treatment is controversial.

**Methods:**

In this retrospective study we evaluated whether antigen‐rapid‐diagnostic‐test (Ag‐RDT) monitoring for SARS‐CoV‐2 in a large cohort of consecutive asymptomatic (absence of SARS‐CoV‐2‐related symptoms such as fever, cough, sore throat or nasal congestion) and fully vaccinated cancer patients enrolled in a short period during cancer treatment has an impact on the therapeutic path of cancer patients.

**Results:**

From December 27, 2021, to February 11, 2022, 2439 cancer patients were screened through Ag‐RDT for SARS‐CoV‐2 before entering the hospital for systemic treatment. Fifty‐three patients (2.17%) tested positive, of whom 7 (13.2%) subsequently developed COVID‐related symptoms, generally mild. Cancer treatment was discontinued, as a precaution, in 49 patients (92.5%) due to the test positivity.

**Conclusion:**

SARS‐CoV‐2 screening in asymptomatic and fully vaccinated cancer patients during systemic treatment appeared to be not cost‐effective: the low rate of SARS‐CoV‐2 positive patients and the low percentage of overt associated infection do not seem proportional to the direct costs (nursing work for swabs, costs of materials and patient monitoring) and indirect costs (dedicated rooms, extension of waiting times for patients and oncologists in delivering therapy as well as its discontinuation in the positive ones). It can, on the other hand, be detrimental when systemic cancer treatment is suspended as a precaution. Given the small number of patients testing positive and the rapid and favorable trend of the infection, it is recommended to always consider continuing systemic oncological treatment, especially when this impacts patient survival as in the adjuvant or neoadjuvant setting.

## Graphical abstract

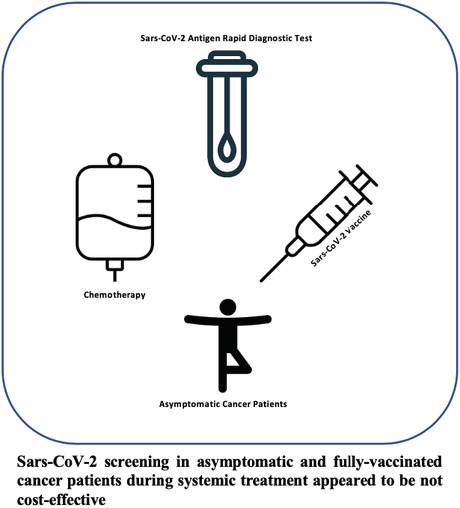

Cancer patients undergoing anti‐cancer treatment were among the first to be vaccinated for SARS‐CoV‐2 in Italy. Although SARS‐CoV‐2 vaccines demonstrated variable rates of seroconversion in subjects with cancer compared to controls,^1^, ^2^, ^3^ antibody response was demonstrated to be elevated after the second and third doses^4^, ^5^, ^6^, ^7^ and the infection rate was lower in vaccinated cancer patients.^8^ However, how clinically meaningful these laboratory findings are is not clear, as studies on clinical efficacy of vaccines are relatively few and mostly retrospective.^9^ A prospective study enrolled 364 cancer patients who received two doses of BBIBP‐CorV COVID‐19 vaccine with the aim of evaluating the proportion of patient developing positive IgG response against SARS‐CoV‐2 Spike protein or neutralizing antibody against Receptor Binding Domain (RBD).^10^ Results demonstrate a high seroconversion rate, particularly in patients younger than 60 years (90% vs. 79% in patients >60 years old, *p* < 0.001), and in patients with solid tumors (94%) compared to those suffering from hematologic malignancies (62% of seroconversion). The antibody response remains high also in patient receiving chemotherapy (83.5%), although lower than in patients treated with endocrine therapy or radiation therapy (97%). A systematic review with meta‐analysis was also recently performed, demonstrating that seroconversion rate after receiving a second dose of COVID‐19 vaccine is high, ranging from 70% in patients with hematologic malignancies to 88% in patients with solid tumors.^11^ In general, vaccination against COVID‐19 in cancer patients has been shown to be safe,^12^ and to provide effective seroconversion.^11^ However, some aspects still need to be clarified, namely the duration of vaccine protection, the type of immune response (humoral and cellular), cancer treatment modalities and their interaction with vaccine immunogenicity.^10^ Routine testing for cancer patients who have been fully vaccinated for SARS‐CoV‐2 prior to systemic treatment who do not present COVID‐19‐related symptoms is a controversial issue. The objective of this retrospective study is to evaluate in a large cohort of a consecutive patients whether screening for SARS‐CoV‐2 in fully‐vaccinated cancer patients undergoing systemic cancer therapy who do not present COVID‐19‐related symptoms influenced, and in which way, their treatment path. Asymptomatic (absence of SARS‐CoV‐2 related symptoms such as fever, cough, sore throat or nasal congestion) and fully vaccinated patients, undergoing cancer systemic treatment for either solid tumor or hematological malignancy were tested and evaluated from December 27, 2021 to February 11, 2022. Ag‐RDT by means of nasal‐swab for SARS‐CoV‐2 was performed by specifically trained nurses in a dedicated spot before patients entered the Institute. A rapid microfluidic‐immunofluorescence assay for the detection of the nucleocapsid protein antigen to SARS‐CoV‐2 (LumiraDX) was used, according to the manufacturer's instructions. If test was negative, patient was allowed admission to the hospital for treatment; in case of positivity, the test was repeated and analyzed individually. If positivity was confirmed, patient was not allowed access to the Institute, and he was remotely monitored by his reference oncologist. We collected characteristics of patients who tested SARS‐CoV‐2 positive, including gender, age, cancer type, treatment setting and type of treatment (Table 1). Direct and indirect costs of all the procedures were considered in order to evaluate the resources used for the screening, among direct costs we considered: the nasal swabs and the immunofluorescence assay kits used to perform the tests. Among the indirect costs we evaluated: the dedicated spaces for testing, waiting times for the patient before being able to access the Institute, waiting times of the oncologist to be able to visit the patient and confirm the planned oncological treatment, the telemedicine follow‐up performed by the oncologist with the positive patient as well as the discontinuation/delay of oncological treatment. This study was evaluated and approved by the local ethics committee (CESC IOV 2022/61).

**TABLE 1:** Characteristics of SARS‐CoV‐2 positive patients

Characteristics	*N* (%); [range; Q1–Q3]
Patients evaluated	2439
SARS‐CoV‐2 positive patients	53 (100)
Gender	
Male	29 (55)
Female	24 (45)
Median age (years)	63 [33–86; Q1: 56‐Q3:72]
Histology	
Gastrointestinal	16 (30.1)
Breast	14 (26.5)
Genitourinary	8 (15.1)
CNS/Head–Neck	5 (9.4)
Hematological	3 (5.7)
Lung	3 (5.7)
Melanoma/Skin	3 (5.7)
Gynecological	1 (1.8)
Setting	
I line systemic treatment	22 (41.5)
≥ II lines systemic treatment	18 (34)
Adjuvant	10 (18.8)
Neoadjuvant	3 (5.7)
Type of treatment	
Chemotherapy	31 (58.7)
Monoclonal antibody	10 (18.8)
Immunotherapy	6 (11)
Target therapy	4 (7.5)
Hormone therapy	2 (4)
SARS‐CoV‐2 Vaccine	
Yes	53 (100)
3 doses	42 (79)
2 doses	11 (21)
SARS‐CoV‐2 patients who have developed symptoms	7 (13.2)
Patients requiring hospitalization for related SARS‐CoV‐2 symptoms	0 (0)
Patients who have delayed cancer therapy due to SARS‐CoV‐2 positivity	49 (92.5)
Median delay in cancer therapy (days)	18.9 [3–46; Q1:14‐Q3:21]

Two thousand four hundred thirty‐nine (2439) cancer patients underwent Ag‐RDT for SARS‐CoV‐2 test before entering the Institute to receive systemic cancer therapy; all patients were vaccinated for SARS‐CoV‐2: 79% had received 3 doses and 21% 2 doses of COVID‐19 BNT162b2mRNA vaccine, with second dose of SARS‐CoV2 vaccine received shortly before Ag‐RDT. Fifty‐three patients (2.17%) tested positive. Fifty‐five percent (55%) were male and median age was 63 years (range 33–86; Q1‐Q3 56–72). The most frequent cancer‐types were gastrointestinal malignancies (30.1%) and breast cancer (26.5%). Forty patients (75.5%) were receiving therapy for advanced/metastatic cancer, and 13 patients (24.5%) were treated in the adjuvant/neoadjuvant setting. Among 53 patients testing positive (2.17% of all screened patients) for SARS‐CoV‐2, only 7 (13.2%) subsequently developed SARS CoV‐2 related symptoms. The most frequently reported related symptoms were fever (71.4%) and cough (57%). The median duration of SARS‐CoV‐2 positivity was 12.6 days (range 3–33), and in 71.4% of positive patient's symptoms were managed at home. Two patients (28.6% of patients who developed COVID‐19 related‐symptoms) required hospital admission to non‐intensive care wards because of cancer related symptoms. Cancer treatment was discontinued in 49 out of 53 (92.5%) SARS‐CoV‐2 positive patients. The median delay in cancer therapy was 18.9 days (range 3–46; Q1‐Q3 14–21), with 71.4% of patients resuming treatment after confirmed SARS‐CoV‐2 swab negativity. An assessment of direct and indirect costs of such monitoring was performed. As direct costs we evaluated: the work of a nurse for nasal swabs, for 5 h/day during the 35 days of analysis (175 total‐hours). The cost of single swabs (2439) and LumiraDX immunofluorescence assay kits capable of analyzing 5 samples simultaneously (approximately 488 kits for total patients). The cost of a second swab analyzed individually must be added to the positive cases to confirm the positivity, for all 5 patients simultaneously analyzed (53 × 5 = 265 additional swabs). Regarding the evaluation of indirect costs, we assessed: the need for a dedicated place, outside the Institute, where staff could safely carry out the tests. Also, such a screening significantly impacted the daily activities of Institute with longer waiting times for both patients and oncologists. A median delay of approximately 1 h for each tested patient (estimate based on the experience of the practitioners involved in the study), with considerable repercussions in the organization of outpatient activities, translating to a median daily delay of an hour and a half for both clinical outpatient visits and administration of chemotherapy, and consequent prolongation of the medical staff and nursing activities.

Our study provides further evidence on Ag‐RDT screening for SARS‐CoV‐2 in a large cohort of fully vaccinated cancer patients undergoing systemic cancer treatment in a Comprehensive Cancer Center not presenting COVID‐19 related symptoms. Several pre‐vaccine reports show a higher risk of mortality for cancer patients infected with SARS‐CoV‐2.^13^, ^14^, ^15^, ^16^, ^17^ More recently, the efficacy of at least two doses of vaccine has been confirmed to prevent the most severe forms of COVID‐19 in cancer patients,^18^ and effective seroconversion in a relevant percentage of cancer patients, even during oncologic treatment has been documented.^19^ Literature data on the rate of seropositivity in patients actively receiving chemotherapy,^20^, ^21^ as well as on the impact of COVID‐19 on anticancer treatment are indeed controversial. Nevertheless, in a meta‐analysis,^22^ chemotherapy performed within 30 days prior to the diagnosis of COVID‐19 did not increase the risk of severe course compared to the control group not receiving chemotherapy(OR:0.92; 95% CI:0.61–1.39) though risk of death from COVID‐19 in the chemotherapy group was higher, after adjusting for confounding variables such as age, sex, cancer type, cancer treatment subtype, duration of cancer diagnosis, smoking status, obesity, performance status, presence of metastases and comorbidities (OR: 1.85; 95% CI:1.26–2.71). The availability of SARS‐CoV‐2 vaccines has significantly decreased the risk of infection and related severe complications in cancer patients,^8^, ^18^ with a safety profile being confirmed in this particular population.^12^ A previous study which tested 537 cancer patients with no COVID‐19 symptoms confirmed a low rate of SARS‐CoV‐2 test positivity (0.64%) as well as a low rate of infection‐related complications in the same group.^23^ Another study compared 150 cancer patients versus 920 non‐cancer patients who had undergone routine test for SARS‐CoV‐2, showing that the cohort of patients with cancer has a lower probability of having a positive test than the control population, leading to the conclusion that routinely screening all cancer patients for SARS‐CoV‐2 may not be worth doing.^24^ The opportunity of continuing cancer treatment despite SARS‐CoV‐2 infection in cancer patients with no COVID‐19‐related symptoms also remains controversial. Results from a German study in which 1227 cancer patients were tested showed that 14 out of 75 asymptomatic SARS‐CoV‐2 positive received chemotherapy, with none of these presenting complications from infection.^25^ In our cohort of 2439 fully vaccinated cancer patients with no SARS‐CoV‐2‐related symptoms screened for SARS‐CoV‐2, 53 patients (2.17%) tested SARS‐CoV‐2 positive and, of these, only 7 (13.2%) developed overt infection with no evidence of severe course and no SARS‐CoV‐2 related deaths. Though the analysis was not performed during one of the major COVID‐19 waves, these data nonetheless confirm a low percentage of positivity, despite a slightly higher rate than previously reported in a smaller group of patients.^23^ Moreover, such a screening procedure in this patient population required the use of considerable resources, albeit for a limited period. The relationship between screening outcomes and both direct and indirect costs is, from our point of view, probably not cost‐effective as it could have a negative impact on cancer patients' therapeutic path. This conclusion may be speculative, as we cannot provide information regarding the effectiveness of the testing; in fact, we cannot have information regarding the outcome of the 53 SARS‐CoV‐2 positive cancer patients if they had not been tested and had continued their oncological treatment without interruption. In an era of health emergency, albeit in a currently less demanding phase, the use of human and material resources should be maximized in the pursuit of cost‐effectiveness and granting the highest level of assistance. Moreover, screening for SARS‐CoV‐2 was associated with discontinuation of oncological treatment in almost all positive patients, with important drawbacks especially in patients undergoing treatment in the (neo)adjuvant and curative setting; failure to guarantee adequate dose intensity, in fact, has well‐known repercussions in terms of mortality in these settings.

The findings of this study have several considerable implications. We found that with proper patient education, vaccination and clinical screening for symptoms or viral exposure, we maintained the outpatient clinical arena largely free of SARS‐CoV‐2,^12^ with good patient's satisfaction.^26^ Moreover, given the rapid and benign evolution of the infection, we recommend considering continuing with systemic anticancer treatment, especially in patients undergoing treatment with curative intent.

In conclusion, screening for SARS‐CoV‐2 in fully‐vaccinated cancer patients without SARS‐CoV‐2 related symptoms during systemic cancer therapy could be considered as a non‐cost‐effective procedure. In this large cohort of cancer patients, the direct and indirect costs required for such screening procedure are high, while numbers of SARS‐CoV‐2 positive patients were low, as well as low was the rate of patients developing SARS‐CoV‐2‐related symptoms. The benefits of such a screening may not offset direct and indirect costs, as no severe course of COVID‐19 course was observed and, on the contrary, could be detrimental due to cancer‐directed treatment interruption.

## AUTHOR CONTRIBUTIONS

**Mario Caccese:** Conceptualization (equal); data curation (equal); investigation (equal); methodology (equal); validation (equal); visualization (equal); writing – original draft (equal); writing – review and editing (equal). **Anna Maria Saieva:** Data curation (equal); investigation (equal); validation (equal); visualization (equal); writing – review and editing (equal). **Valentina Guarneri:** Data curation (equal); investigation (equal); validation (equal); visualization (equal); writing – review and editing (equal). **Sara Lonardi:** Data curation (equal); investigation (equal); validation (equal); visualization (equal); writing – review and editing (equal). **Massimo Cacco:** Data curation (equal); investigation (equal); validation (equal); visualization (equal); writing – review and editing (equal). **Vanna Chiarion Sileni:** Data curation (equal); investigation (equal); validation (equal); visualization (equal); writing – review and editing (equal). **Michele Gottardi:** Data curation (equal); investigation (equal); validation (equal); visualization (equal); writing – review and editing (equal). **Eleonora Mioranza:** Data curation (equal); investigation (equal); validation (equal); visualization (equal); writing – review and editing (equal). **Francesca Bergamo:** Data curation (equal); investigation (equal); validation (equal); visualization (equal); writing – review and editing (equal). **Antonella Brunello:** Conceptualization (equal); data curation (equal); formal analysis (equal); investigation (equal); methodology (equal); supervision (equal); validation (equal); visualization (equal); writing – original draft (equal); writing – review and editing (equal). **Vittorina Zagonel:** Conceptualization (equal); data curation (equal); formal analysis (equal); investigation (equal); methodology (equal); supervision (equal); validation (equal); visualization (equal); writing – original draft (equal); writing – review and editing (equal). **Patrizia Benini:** Data curation (equal); investigation (equal); validation (equal); visualization (equal); writing – review and editing (equal).

## FUNDING INFORMATION

This study was supported by “Ricerca Corrente 2022 of the Italian Ministry of Health”.

## CONFLICT OF INTEREST

All authors declare no conflicts of interest.

## Data Availability

Data available on request due to privacy/ethical restrictions.
